# Benefits of Integrating Telemedicine and Artificial Intelligence Into Outreach Eye Care: Stepwise Approach and Future Directions

**DOI:** 10.3389/fmed.2022.835804

**Published:** 2022-03-11

**Authors:** Mark A. Chia, Angus W. Turner

**Affiliations:** ^1^Lions Outback Vision, Lions Eye Institute, Nedlands, WA, Australia; ^2^Institute of Ophthalmology, Faculty of Brain Sciences, University College London, London, United Kingdom; ^3^Centre for Ophthalmology and Visual Science, University of Western Australia, Nedlands, WA, Australia

**Keywords:** telemedicine, ophthalmology (MeSH), rural health services, indigenous health services, quality of health care (MeSH), artificial intelligence

## Abstract

Telemedicine has traditionally been applied within remote settings to overcome geographical barriers to healthcare access, providing an alternate means of connecting patients to specialist services. The coronavirus 2019 pandemic has rapidly expanded the use of telemedicine into metropolitan areas and enhanced global telemedicine capabilities. Through our experience of delivering real-time telemedicine over the past decade within a large outreach eye service, we have identified key themes for successful implementation which may be relevant to services facing common challenges. We present our journey toward establishing a comprehensive teleophthalmology model built on the principles of collaborative care, with a focus on delivering practical lessons for service design. Artificial intelligence is an emerging technology that has shown potential to further address resource limitations. We explore the applications of artificial intelligence and the need for targeted research within underserved settings in order to meet growing healthcare demands. Based on our rural telemedicine experience, we make the case that similar models may be adapted to urban settings with the aim of reducing surgical waitlists and improving efficiency.

## Introduction

The delivery of equitable eye services for rural and remote communities represents a unique challenge to healthcare providers. Within Western Australia (WA), the integration of teleophthalmology into service delivery has played a pivotal role in addressing these challenges. Lions Outback Vision was established in 2010 at the Lions Eye Institute in Perth and now serves 51 communities with visiting optometry and/or ophthalmology. This article presents an overview of our journey toward the development of an integrated teleophthalmology model over the past decade, with a focus on the key lessons for building an effective telemedicine service. Beyond telemedicine, we consider the role of recent advancements in artificial intelligence (AI) and the pathway toward harnessing this technology for more equitable service provision in under-resourced settings. Finally, we make the case that outreach telemedicine models may be translated into urban areas to address the problem of burgeoning surgical waitlists. The coronavirus 2019 (COVID-19) pandemic has accelerated telemedicine capabilities across the globe and catapulted its applications beyond traditional geographic barriers to healthcare.

## Telemedicine Integration for Outreach Eye Care

With an area of 2.65 million square kilometers, the state of WA would feature within the top 10 countries by size worldwide. Ninety percent of the population live within the southwest corner of the state, centered around the capital city where all tertiary services are located. The remaining population is scattered sparsely across outback WA, representing a significant challenge for eye care providers. Remote health services are frequently affected by high staff turnover, impacting on long-term stability. Furthermore, rural areas have a high proportion of Indigenous patients compared to metropolitan areas. Patient rurality and Indigenous status are both associated with a higher burden of vision impairment coupled with reduced access to eye care services (1).

**Figure 1:**
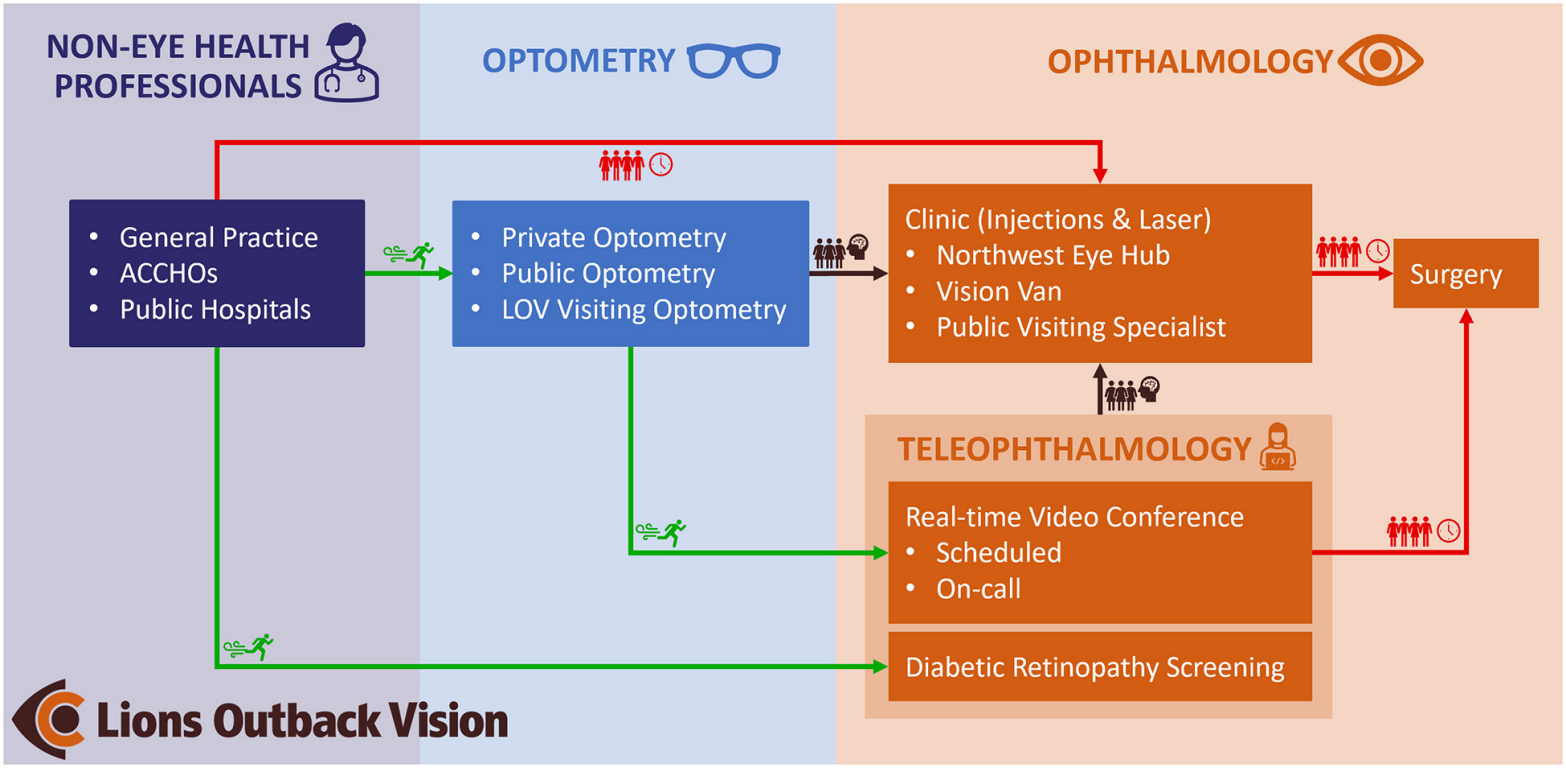
Clinical pathway demonstrating the role of teleophthalmology within Lions Outback Vision. Teleophthalmology enables fast-track access (green) for patients to specialist care compared to traditional referral pathways which are congested (red). By diverting referrals from non-eye care professionals via optometry and diabetic retinal screening, specialist clinics can manage well-triaged pathology (brown). ACCHOs, Aboriginal Community Controlled Health Organizations; LOV, Lions Outback Vision.

Given the unique demography of WA, teleophthalmology has been a key and growing service element within Lions Outback Vision through both real-time videoconferencing and “store and forward” modalities (Figure 1). In 2021, 25% (*n* = 1,825) of all ophthalmology appointments at our service were conducted though telemedicine. During face-to-face outreach specialist visits, 62% (*n* = 3,442) of appointments required specialist procedural management, representing a highly efficient clinical triage through collaboration with optometrists. The ability to waitlist surgical patients via videoconference at the time of primary-care assessment eliminates the waiting time for the initial specialist appointment with attendant logistical and cost implications. Moreover, teleophthalmology can also be delivered safely utilizing the correct expertise and case-selection, with a systematic review finding that diagnostic accuracy for real-time teleophthalmology was comparable to face-to-face consultation (2).

We believe that our experience in delivering real-time videoconference consults over the last decade may provide useful lessons for similar regions around the world. Through our history of service delivery, we have identified several key lessons in our journey. These include: 1) a focus on coordination of services at both regional and local community levels, 2) engagement with government funding agencies to align telemedicine-related financial incentives with the benefits they deliver, and 3) reducing barriers to telemedicine uptake through a range of service modifications, education, and support initiatives.

### Coordination of Eye Services

Coordination between ophthalmology and optometry has been identified as an essential part of delivering effective outreach eye care. A cross-sectional case study of rural eye services in Australia demonstrated that higher levels of integration between optometrists and ophthalmologists led to improved surgical case rates, with trends toward increased clinical activity and reduced wait times (3). The primary screening and triage provided by optometrists funneled more patients with a higher concentration of pathology requiring procedural intervention to the limited number of specialist visits. Important elements of coordination were highlighted including: 1) service integration with optometry services to facilitate primary screening and triage, 2) involvement of local health staff such as Aboriginal Health Workers to support patient attendance, and 3) appointment of a Rural Eye Health Coordinator (REHC) to liaise between primary healthcare, regional hospitals, and visiting eye services.

In 2011, Lions Outback Vision implemented real-time videoconference teleophthalmology services that linked patients to an ophthalmologist and was facilitated by their primary healthcare provider. To build an evidence base, we conducted a series of studies designed to evaluate our service and found that several of the themes highlighted above regarding eye service coordination were also critical for telemedicine. A prospective clinical audit of 100 telemedicine consultations showed that 60% of referrals emanated from optometrists, despite there being no reimbursement for referral at that time, and the remainder of the referrals were generated from general practitioners (4). A survey of 109 patients who took part in telemedicine consultations found a high level of satisfaction, with 94% of patients indicating they were “satisfied” or “very satisfied” (5). Qualitative analysis of the factors contributing to satisfaction revealed that familiarity with staff at the patient-end was important, in part making up for any perceived impersonality due to the absence of face-to-face interaction. This again highlights the essential part that local community staff play in facilitating effective teleophthalmology.

The key role of REHCs has been demonstrated within our diabetic retinopathy screening program, which operates using a store-and-forward model (6). Following a period of declining screening activity in WA's Kimberley region, an REHC was appointed with the aim of providing high-level support for retinal screening and staff training. A retrospective audit comparing the period before and after the REHC's appointment showed an increase in screening coverage from 9 to 30%, with the number of screening sites increasing from 4 to 17 (6). This illustrates the positive impact that regional coordination can have on the effective delivery of teleophthalmology.

### Alignment of Funding Incentives

A high degree of engagement with government funding bodies has been critical to the success of our telemedicine program, with the aim of ensuring that reimbursement sustains services and reflects the costs of high-quality service delivery. In 2011, the Commonwealth government introduced Medicare funding for both the referring doctor and specialist, with ~50% loading above equivalent face-to-face visits to reflect the additional resources required for telemedicine consults. Limited reimbursement in the United States is a frequently cited reason for reduced uptake of teleophthalmology (7). In 2019, only 10 of 50 states had payment parity between telemedicine and office visits (7). In response to COVID-19, telemedicine was made more widely available through reimbursement at the same rate as in-person visits regardless of setting. Similarly, in Australia regulations have been temporarily relaxed to include funding for audio-only consultations as well as metropolitan settings.

Despite the introduction of sustainable funding, telemedicine uptake in WA was initially low, falling 74% below government targets in the first year that incentives became available (8). Our group conducted an analysis of structural and economic drivers within WA eye care services with the aim of increasing the impact of teleophthalmology in Australia (9). Based on clinical audits of 5,456 eye visits, an estimated 15% of urgent transfers and 24% of outreach consultations were assessed as being suitable for telemedicine, leading to an estimated annual cost-saving of $1.1 million. Additionally, to determine the initial capital expenditure required to facilitate basic teleophthalmology, we conducted a survey of ocular diagnostic and teleconference equipment available at optometrists, primary-care practices, and hospitals. Setup costs for primary-care practices were estimated at $20,500, compared to negligible costs for already well-equipped optometry practices.

Based on this finding, along with the reduced need for further eye-specific clinical training for optometrists, we concluded that facilitating optometrist-led teleophthalmology provided the most compelling economic case. A strategy document was created with recommendations for teleophthalmology in Australia and submitted to the Royal Australian and New Zealand College of Ophthalmologists and Optometry Australia. Amongst other strategic initiatives, successful advocacy resulted in the approval of new Medicare codes for optometrist-led rural telemedicine referrals in 2015. In the first year following approval, Lions Outback Vision received 709 teleophthalmology referrals facilitated by optometrists (10).

### Addressing Barriers to Telemedicine Uptake

Despite the demonstrated benefits of telemedicine, there are numerous barriers that can limit widespread uptake. In addition to the financial barriers previously outlined, there are also a range of technical and logistical obstacles. Taking a proactive approach to addressing these challenges has been critical to developing our service model. We found that introducing a multi-faceted intervention increased teleophthalmology uptake at our service (11). Key elements of this intervention included awareness raising, educational resources, logistical support, an updated online booking system, and a funding mechanism to simulate Medicare payments prior to government implementation.

In the United States, restrictions on permitted videoconferencing software emerged as an important barrier to telemedicine uptake in the context of COVID-19 (7). In response, the Centers for Medicare and Medicaid Services removed penalties for technologies that were previously considered non-compliant, such as Facetime, Skype, and Google Hangout. In contrast, these types of technologies have been used for telemedicine in Australia since 2011. Our existing service remains agnostic to videoconference platforms in order to minimize barriers for the referrer. Multiple audits of our service have shown that freely available voice-over-Internet-Protocol services such as Skype and Facetime are commonly chosen by referrers, supporting the idea that familiarity and useability are critical factors (10, 11).

Scheduling three parties (patient, referrer and specialist) for synchronous telemedicine relies on availability and timeliness of participants to ensure minimal disruption to clinical workflow. In 2017, our service introduced on-call teleophthalmology services to complement the existing online booking system. When an optometrist is visiting a community or a patient has traveled a significant distance for an assessment, there is limited opportunity for a scheduled future telemedicine appointment. This alternative provided immediate access to the ophthalmologist, resulting in improved access for rural and Indigenous patients. A clinical audit showed that the proportion of Indigenous patients in the on-call telemedicine cohort was 51.4%, compared to 8.7% in the online-booking telemedicine group (12). We found similar improvements in access for the most remote regions of WA, with 79.0% in the on-call service compared to 26.1% in the online-booking cohort. Of all telemedicine consultations in 2018, 27.8% made use of the on-call service, demonstrating high demand for the more flexible booking arrangement.

## Beyond Telemedicine: The Role of AI in Rural Eye Care

Rapid progress in AI technology has attracted interest due to its potential to perform complex medical tasks with supra-human performance. Deep learning is a type of AI utilizing multiple processing layers to learn representation of data with multiple levels of abstraction (13). Deep learning is particularly well-suited to image analysis tasks; hence, image-driven specialties like ophthalmology have become the frontrunners for medical AI. Advances in AI hold promise in helping bridge the widening gap between population eye care needs and trained human resources to meet these needs, particularly for underserved rural communities. A well-known limitation of AI is the tendency for algorithms to generalize poorly outside their research milieu (14). AI systems must therefore be trained on data from diverse populations and then rigorously validated within their intended settings.

### Autonomous Diabetic Retinopathy Screening

Ophthalmic AI applications exist for numerous conditions, most commonly for diabetic retinopathy (DR), glaucoma, and age-related macular degeneration (AMD) (13). DR represents a growing burden for outreach eye services due to increasing prevalence, requirement for expensive imaging equipment, and the need for regular specialist intervention for optimal outcomes. It is therefore reassuring that much of the most promising progress toward real-world AI implementation has been for autonomous DR screening. Currently, two autonomous DR screening systems have been approved for use by Food and Drug Administration, following pivotal trials showing strong performance in real-world settings (15, 16). Both studies were conducted in the United States within majority white populations.

Our group was recently involved with a real-world validation study of a separate autonomous DR screening system evaluating 236 diabetic patients from Aboriginal Medical Services and endocrinology outpatient clinics (17). In addition to identifying referable DR, the system was also designed to screen for AMD and glaucoma, although performance was only assessed for DR screening. The system achieved a sensitivity and specificity of 96.9 and 87.7%, respectively, in detecting referable DR. Apart from investigating an at-risk ethnic group, other novel aspects of the study included: 1) the use of an offline AI system rather than a cloud-based system, as the latter is a notable barrier in some rural settings, 2) use of several types of retinal camera, and 3) evaluation of patient and clinician acceptability, which is frequently cited as a major limitation to AI uptake. Of 207 participants who completed a satisfaction questionnaire, 93.7% stated that they were either “satisfied” or “extremely satisfied”. Clinicians most frequently noted that the AI system was easy to use, and that the real-time diagnostic report was helpful.

### Research Potential at Australia's First Remote Eye Center

Despite the progress of ophthalmic AI applications, further study is required to ensure that AI technology delivers benefit to patients. In 2021, Lions Outback Vision opened the Northwest Eye Hub in the remote Kimberley town of Broome. As Australia's first permanent dedicated eye center located in a remote region, it holds significant potential for furthering AI research in this high-risk but under-studied population. Our decade-long history of working in partnership with local community leaders and health organizations means we are well-positioned to develop further collaborative research partnerships. The hub is equipped with state-of-the-art diagnostic equipment, which, in many cases, surpasses that of tertiary eye clinics. The center is staffed by two full-time ophthalmologists and a range of other health staff including Aboriginal Health Workers and optometrists. Our team is currently engaged in several AI projects focusing on DR screening, detecting macular edema, (18) and analyzing optical coherence tomography angiography linking systemic risk factors.

## Translational Telemedicine: Outback Solutions to Big City Problems

A significant “hidden” waitlist has been recently highlighted in Australia—the waiting time for initial specialist assessment (19). This pre-specialist assessment waiting time is often not publicly available and yet masks a burden of preventable diseases silently resulting in unknown levels of permanent blindness or unnecessarily prolonged visual impairment. An audit of cataract referrals from two metropolitan public hospitals in New South Wales found that two-thirds of patients were yet to have their initial hospital appointment in the year following referral (20).

The COVID-19 pandemic has elevated telemedicine in the consciousness of all health service providers attempting to bridge barriers to healthcare. The imperative for telemedicine in outback Australia over the last decade and the robust supporting evidence-base can be translated rapidly to urban settings. For eye care in Australia, optometrists represent an accessible, publicly funded, and well-equipped resource to help tackle population eye health needs. Collaborative care models involving community-based optometrists and virtual review by an ophthalmologist using “store-and-forward” telemedicine modalities have been demonstrated for glaucoma and diabetes clinics in Australia, leading to cost-savings and reduced wait times (21, 22). Exploring options for upscaling these models has the potential to further improve the capacity of public eye services.

Synchronous videoconferencing may also be utilized to consent patients for surgical management during their first in-person contact point with the specialist, as shown within Lions Outback Vision. An audit of outreach surgery found that patients assessed through telemedicine waited half the length of time compared to those assessed in traditional outpatient clinics (23). Urban centers in the United Kingdom have explored comparable models involving community optometrists and telemedicine consultations to enable “one stop cataract surgery,” demonstrating similar benefits (24, 25). Adapting these models to the Australian context will require careful consideration to safeguard informed consent, rigorous surgical risk-assessment, and effective use of theater-time, however the benefits warrant further exploration. Telemedicine provides a seamless path from primary care to surgical management, and enables expert medical input where required, establishing a cornerstone to collaborative care.

## Conclusion

Within WA, integration of teleophthalmology has been a crucial component in enabling Lions Outback Vision to make progress toward equitable eye care delivery. Much of this headway has relied upon establishing collaborative care models with regional optometrists, maximizing the efficiency of in-person specialist visits. Key lessons from our service have the potential to be applied to areas that share similar geographical and logistical challenges. Looking forward, advances in AI have shown promise toward bridging the gap between expanding eye care demands and limited resources; however, further investigation within under-resourced settings is critical to future progress. Finally, following the acceleration of global telemedicine capabilities triggered by COVID-19, lessons from rural services may be applied to urban centers to curb rapidly growing surgical wait lists. There is a clarion call to harness telemedicine advances in collaborative care to preserve sight in both urban and rural settings.

## Data Availability Statement

The original contributions presented in the study are included in the article/supplementary material, further inquiries can be directed to the corresponding author.

## Author Contributions

MC and AT were involved in planning, researching, and drafting the manuscript. The final manuscript was reviewed and approved by both authors.

## Funding

MC is completing a PhD at University College London funded by a General Sir John Monash Scholarship.

## Conflict of Interest

The authors declare that the research was conducted in the absence of any commercial or financial relationships that could be construed as a potential conflict of interest.

## Publisher's Note

All claims expressed in this article are solely those of the authors and do not necessarily represent those of their affiliated organizations, or those of the publisher, the editors and the reviewers. Any product that may be evaluated in this article, or claim that may be made by its manufacturer, is not guaranteed or endorsed by the publisher.
